# A Review of the Current Literature Regarding Sodium-Glucose Cotransporter 2 Inhibitors in Heart Failure With Preserved Ejection Fraction and Timing of Initiating Therapy

**DOI:** 10.7759/cureus.113821

**Published:** 2026-08-02

**Authors:** Mark Sahyouni, Chirag Lodha, Vivek Joseph Varughese

**Affiliations:** 1 Internal Medicine, Prisma Health/University of South Carolina School of Medicine, Columbia, USA; 2 Internal Medicine, University of South Florida Morsani College of Medicine, Tampa, USA

**Keywords:** cardiology, heart failure with preserved ejection fraction, heart failure with reduced ejection fraction, hfpef, hfref, sglt 2 inhibitor, sodium glucose cotransporter 2 inhibitors (sglt2i)

## Abstract

Heart failure with preserved ejection fraction (HFpEF) is a growing public health burden that now accounts for nearly half of all HF diagnoses in the United States. Modern classifications of HF divide patients into HFpEF and HF with reduced ejection fraction (HFrEF). Historically, diagnosis has relied on the Framingham Heart Failure Diagnostic Criteria, which incorporates clinical signs and symptoms including pulmonary edema, jugular venous distention, orthopnea, S3 gallop, cardiomegaly, peripheral edema, dyspnea on exertion, and tachycardia. Contemporary definitions of HF further incorporate objective evidence such as elevated natriuretic peptide levels, cardiopulmonary congestion, and echocardiographic findings including preserved left ventricular ejection fraction and evidence of diastolic dysfunction. HFpEF predominantly affects older adults and is strongly associated with hypertension, obesity, diabetes mellitus, coronary artery disease, atrial fibrillation, and chronic kidney disease. Despite advances in management, HFpEF continues to be associated with substantial morbidity, mortality, recurrent hospitalization, and healthcare utilization.

Therapeutic options for HFpEF have historically been limited, with few interventions demonstrating meaningful reductions in hospitalization or cardiovascular mortality. However, sodium-glucose cotransporter 2 inhibitors (SGLT2i) have emerged as a promising cornerstone of therapy. Initially developed for glycemic control in patients with diabetes mellitus, SGLT2i have demonstrated broad cardiovascular and renal benefits extending beyond glucose lowering. Recent large randomized controlled trials evaluating empagliflozin and dapagliflozin in patients with HFpEF have shown significant reductions in HF hospitalizations and improved cardiovascular outcomes across diverse patient populations, including those without diabetes mellitus. These findings have substantially influenced contemporary guideline-directed medical therapy recommendations. Current American and European HF guidelines now support the use of SGLT2i in HFpEF, with European recommendations assigning the highest level of recommendation and evidence for their routine use in eligible patients. Contraindications to therapy remain limited and include type 1 diabetes mellitus, end-stage renal disease requiring dialysis, and pregnancy or lactation.

Although evidence supporting SGLT2i use in HFpEF continues to strengthen, uncertainty remains regarding the optimal timing of therapy initiation. In particular, there is ongoing debate surrounding whether initiation during hospitalization for acute HF exacerbation confers greater clinical benefit compared with delayed outpatient initiation after discharge. Early inpatient initiation may improve medication adherence, reduce rehospitalization rates, accelerate symptomatic improvement, and optimize implementation of guideline-directed medical therapy. Conversely, concerns regarding hemodynamic stability, renal function, volume status, and transitional care logistics continue to influence clinician decision-making in the inpatient setting. This literature review aims to explore the current literature regarding SGLT2i in HFpEF, as well as the optimal time to initiate therapy.

## Introduction and background

Modern definitions of heart failure (HF) are categorized into two distinct categories: HF with preserved ejection fraction (HFpEF) and HF with reduced ejection fraction (HFrEF). Diagnosis of HF is centered around the Framingham HF Diagnostic Criteria developed in 1971 [1-2]; this includes signs and symptoms of two major criteria or one major criterion and two minor criteria. The major criteria include acute pulmonary edema, jugular venous distention, orthopnea, S3 gallop, cardiomegaly, and/or hepatojugular reflux. The minor criteria include peripheral edema, pleural effusion, dyspnea on exertion, nocturnal cough, and/or tachycardia >120 beats per minute [1-2]. The newer definition of HF diagnosis is based on signs and symptoms consistent with HF per the Framingham criteria, in addition to elevated natriuretic peptides and/or evidence of cardiopulmonary congestion [2]. Additional objective information of an ejection fraction >50% on echocardiogram with evidence of diastolic dysfunction also supports the diagnosis [2-3]. 

HFpEF affects approximately three million people in the United States, representing about 50-60% of all HF cases, and the large majority of HF patients [2]. It is estimated that the lifetime risk of HFpEF is ~10% beginning at age 45 years in both men and women [4]. Risk factors for HFpEF include hypertension, obesity, diabetes, coronary artery disease, atrial fibrillation, and chronic kidney disease [5]. Management of HFpEF is evolving, with new trials suggesting that certain subgroups of guideline-directed medical therapy (GDMT) are more beneficial than others. Sodium-glucose cotransporter 2 inhibitors (SGLT2i) currently have a class IIa recommendation in the 2023 ACC guidelines [5]. The 2023 European Society of Cardiology (ESC) Heart Failure Guideline update gives SGLT2i use in HFpEF a Class I (Level A) recommendation, the strongest possible recommendation [6]. Several new trials have emerged since that suggest SGLT2i are gaining traction for use in HFpEF with even stronger levels of evidence to suggest all HFpEF patients should be on them, with the absence of contraindications. Contraindications include type I diabetes, end-stage renal disease requiring dialysis, and lactation [5]. Major landmark trials such as the empagliflozin outcome trial in patients with chronic HF with preserved ejection fraction (EMPEROR-preserved) and dapagliflozin evaluation to improve the lives of patients with preserved ejection fraction HF (DELIVER) support guidelines in support of SGLT2i use in HFpEF. The ESC’s guidelines are centered around these trials and allude to the difference in levels of recommendation. 

Even with stronger levels of evidence emerging for the use of SGLT2i in HFpEF, a gap in evidence in guidelines exists on optimal initiation. There is debate about whether the medication should be started inpatient as opposed to outpatient during follow-up after hospitalizations for HF exacerbations. This review aims to summarize current evidence regarding optimal timing of the use of SGLT2i and whether its inpatient initiation makes an impact on morbidity and mortality in HF patients. 

## Review

Methods

Study Design

SGLT2i block glucose and sodium reabsorption in the proximal tubule of the kidney and promote osmotic diuresis and natriuresis [7-9]. They primarily reduce interstitial fluid while preserving plasma volume, unlike conventional loop diuretics [10]; the primary mechanism is through enhanced capillary permeability and lymphatic drainage, all while alleviating myocardial stiffness and pulmonary congestion [10]. The diuretic effect reduces plasma volume and therefore has a reduction in preload and afterload, which helps treat HF [8]. A 3-5 mmHg reduction in arterial pressure has been measured with the use of SGLT2i, therefore reducing arterial stiffness [9]. SGLT2i enhances hemodynamic changes that limit congestion and promote cardiac remodeling [8]. They have also been shown to reduce the amount of sodium in the skin by 23-Na magnetic resonance imaging; this sodium reduction could potentially reduce the risk of left ventricular hypertrophy and improve left ventricular remodeling [9].

Oxidative stress generated via reactive oxygen species causes chronic damage in patients with HF via mitochondrial damage, sequestration of nitric oxide, and matrix degradation [7]. SGLT2i has also been shown to reduce biomarkers associated with oxidative stress in as little as a few weeks [7]. Biomarkers specific to oxidative stress are 8-isoPGF2a and 8-OHdG [7]. They also help minimize oxidative stress and inflammation by modulating cytokine production [8]. SGLT2i directly attenuates oxidative stress through decreased expression of nicotinamide adenine dinucleotide phosphate (NADPH) oxidases and alleviation of mitochondrial injury, both of which reduce the excessive generation of reactive oxygen species that damage endothelial cells [11]. Empagliflozin has been shown to lower circulating TNF-a, IL-1B, and NLRP3, all of which are drivers of inflammation and ventricular dilation and HF [7,9]. 

Another compounding effect that worsens HF over time is vascular changes that disrupt endothelial function via arterial stiffness and impaired local oxidative and inflammatory signals [7-8]. SGLT2i supports endothelial function via greater nitric oxide bioavailability, which reduces arterial stiffness, medial thickening, and collagen deposition [7]. The gasotransmitter nitric oxide is enhanced by SGLT2i via the PI3K/Akt/eNOS axis, which restores endothelial homeostasis in patients with HF [11]. Simultaneously, they will suppress inflammation-related signaling through inhibition of the NF-kB pathway, a pathway that contributes to the expression of adhesion molecules and proinflammatory cytokines; inhibition of this pathway reduces antagonistic vascular remodeling [11]. An additional mechanism contributing to endothelial homeostasis includes the inhibition of overactive sodium-hydrogen exchangers, leading to normalization of intracellular sodium and calcium concentrations that influence endothelial cell metabolism and function [11].

Key clinical trials

Two major landmark trials support the use of SGLT2i in patients with HFpEF: EMPEROR-preserved and DELIVER. In EMPEROR-preserved, the trial enrolled nearly 6,000 patients with HFpEF and demonstrated a 21% reduction in the primary composite outcome of cardiovascular death or HF hospitalization with empagliflozin 10 mg daily (hazard ratio (HR): 0.79; 95% confidence interval (CI): 0.69 to 0.90; p < 0.001) [12]. In secondary outcomes measured, there was a statistically significant reduction in hospitalizations on SGLT2i, which the authors synthesized as the main determinant of reduced cardiovascular death (HR: 0.73; 95% CI: 0.61 to 0.88; p < 0.001) [12]. There was no statistically significant difference in cardiovascular death among patients treated with empagliflozin and placebo (HR: 0.91 (95% CI: 0.76-1.09) [12]. The number needed to treat (NNT) was 31 (95% CI: 20-69) over 26 months [12]. There was also an improvement seen in the Kansas City Cardiomyopathy Questionnaire (KCCQ) score after 52 weeks (difference: 1.32; 95% CI: 0.45-2.19) [12]. The KCCQ is a subjective scoring assessing the patients’ frequency and severity of symptoms, physical limitations, quality of life, social limitations, and self-efficacy [13]. 

Similarly, DELIVER is a similar-style randomized controlled trial (RCT) that enlisted nearly 6,000 patients demonstrating an 18% reduction in the primary composite outcome of cardiovascular death or worsening HF with dapagliflozin 10 mg daily (HR: 0.82; 95% CI: 0.73 to 0.92; p < 0.001) [14]. HF hospitalization occurred in 10.5% of the dapagliflozin group versus 13.3% of the placebo group (HR: 0.77; 95% CI: 0.67-0.89) [14]. The absolute event rates for the primary outcome for those on dapagliflozin (16.4%) vs. placebo (19.5%) yielded an absolute risk reduction of 3.1% (HR: 0.82; 95% CI: 0.73-0.92; p < 0.001) [14]. The trial observed less worsening HF in the treatment arm compared to placebo (HR: 0.39; 95% CI: 0.69-0.91) [14]. There was less cardiovascular death observed in the treatment group (7.4%) compared to placebo (8.3%) (HR: 0.88; 95% CI: 0.74 to 1.05) [14]. Other secondary outcomes measured saw less worsening of HF in the treatment group (95% CI: 0.67-0.89; p < 0.001) [14]. The KCCQ score was on average 2.4 points higher while on dapagliflozin (95% CI: 1.5-3.4) (Table 1) [14]. 

**Table 1:** Outpatient trials for SGLT2i initiation SGLTi: sodium glucose transporter-2 channel inhibitors

Trial	Anker et al. [12]	Solomon et al. [14]
Population and intervention	5,888 patients; empagliflozin vs. placebo; median follow-up: 26 months	6,263 patients; dapagliflozin vs. placebo; median follow-up: 27.6 months
Primary Outcomes	21% reduction in the primary composite outcome of cardiovascular death or heart failure hospitalization with empagliflozin 10 mg daily (HR: 0.79; 95% confidence interval (CI): 0.69 to 0.90; p < 0.001)	18% reduction in the primary composite outcome of cardiovascular death or worsening heart failure with dapagliflozin 10 mg daily (HR: 0.82; 95% CI: 0.73 to 0.92; p < 0.001)
Secondary outcomes	Reduction in hospitalizations (HR: 0.73; 95% CI: 0.61 to 0.88; p < 0.001)	Reduction in hospitalizations (HR: 0.77; 95% CI: 0.67-0.89)
	No statistically significant difference in cardiovascular death (HR: 0.91; 95% CI: 0.76-1.09)	No statistically significant difference in cardiovascular death (HR: 0.88; 95% CI: 0.74 to 1.05)
	NNT was 31 (95% CI: 20-69) over 26 months	NNT was 32 (95% CI: 0.73-0.92) over 27 months
	KCCQ score +1.32 (95% CI: 0.45-2.19)	The KCCQ score +2.4 (95% CI: 1.5-3.4)

The sotagliflozin in patients with diabetes and recent worsening leading to increased stress in treatment-worsening heart failure (SOLOIST-WHF) trial was a multicenter, double-blinded study looking at ~1200 patients with type 2 diabetes recently hospitalized with HF randomly assigned to receive sotagliflozin or placebo. Nearly half of the cohort received sotagliflozin prior to discharge and the other cohort within two days after discharge [15]. The median follow-up was nine months. Approximately 21% of these patients had HFpEF. The researchers looked at the total number of cardiovascular deaths and HF-related hospitalizations for their primary outcomes [15]. The primary endpoint was reached at a significantly lower rate while on sotagliflozin compared to placebo (HR: 0.67 (95% CI: 0.52-0.85); p < 0.001) [15]. This significance is likely attributed to a decrease in repeat hospitalizations and urgent visits, not mortality, as seen in the secondary outcome. Secondary outcomes included were total hospitalizations (194) and urgent visits (297) (HR: 0.64 (95% CI: 0.49-0.83), p < 0.001), cardiovascular death (95% CI: 0.58-1.22; p = 0.36), KCCQ-12 score after four months (95% CI: 1.3-7.0; p = 0.005), and change in eGFR (95% CI: -1.30 to 0.98) [15].

Empagliflozin in patients hospitalized with acute HF who have been stabilized (EMPULSE) was a double-blinded, multinational RCT that evaluated the effect of empagliflozin vs placebo in hospitalized individuals with acutely decompensated HF; approximately 22% had an LVEF of >50% [16-17]. They enrolled 530 patients, and the median follow-up was 90 days [16]. In the study, empagliflozin was initiated within three days of patient stabilization and continued up to 90 days [16]. The study’s primary endpoint was a composite of all-cause death, HF-related events, time to first HF event, and ≥5-point change in KCCQ total symptom score [16]. The trial demonstrated significant clinical improvement on empagliflozin vs. placebo (stratified win ratio: 1.36; 95% CI: 1.09-1.68; p = 0.0054). Secondary outcomes in the trial included the incidence of cardiovascular deaths or HF exacerbations with (12.8%) and without (18.5%) empagliflozin (HR: 0.69; 95% CI: 0.45-1.08) [16]. Patients treated with empagliflozin had a greater change in KCCQ compared to those on placebo after 90 days (adjusted mean difference: 4.45 points; 95% CI: 0.32-8.59), but there was no significant difference in the proportion of patients who improved by 10 points on the KCCQ [16]. There was a statistically significant drop in NT-proBNP in the treatment group compared with placebo at day 30 (adjusted geometric mean ratio: 0.90; 95% CI: 0.82-0.98) [16]. A dedicated LVEF-stratified analysis of EMPULSE by Tromp et al. evaluated outcomes across the EF spectrum and found comparable findings; EF <40% win ratio 1.35 (95% CI: 1.04, 1.75); EF 41-49% win ratio: 1.25 (0.66, 2.37) and EF >50% win ratio: 1.40 (0.87, 2.23, pinteraction = 0.96) [17]. Their analysis showed the primary endpoint was not modified based on treatment status or LVEF [17]. It also did not show a difference in all-cause mortality, KCCQ, or HF exacerbations (ptrend > 0.05 for all) [17]. The risk of cardiovascular death or HF exacerbation was lower in the treatment group in the first three months regardless of LVEF, but was not statistically significant (ptrend = 0.35) [17].

Dapagliflozin and effect on cardiovascular events in acute heart failure-thrombolysis in myocardial infarction 68 (DAPA ACT HF-TIMI 68) is a randomized, double-blinded, placebo-controlled trial published in 2025 that evaluated in-hospital initiation of dapagliflozin in 2,401 patients hospitalized for HF. Of note, patients enrolled in the study exhibited HFrEF with an LVEF of ≤40%: 71.5% (1,717 patients), and the remaining 28.5% of patients had an LVEF of >40% [18]. The primary outcome was a composite of time to cardiovascular death or worsening HF through two months, which found no statistically significant difference in patients with or without dapagliflozin (0.86; 95% CI: 0.68-1.08; p = 0.20) [18]. Other findings include worsening HF events: 9.4% vs. 10.3% (HR: 0.91; 95% CI: 0.71-1.18); cardiovascular death: 2.5% vs. 3.1% (HR: 0.78; 95% CI: 0.48-1.27); and all-cause death: 3.0% vs. 4.5% (HR: 0.66; 95% CI: 0.43-1.00) [18]. The rates of symptomatic hypotension were 3.6% and 2.2%, and the rates of worsening kidney function were 5.9% and 4.7% with dapagliflozin and placebo, respectively [18].

A prespecified meta-analysis combining the data from DAPA ACT HF-TIMI 68, EMPULSE, and SOLOIST-WHF showed significant improvement in outcomes in the SGLT2i treatment group when initiated inpatient. This includes a 29% reduction in cardiovascular death and worsening HF (HR: 0.71 (95% CI: 0.54-0.93; p = 0.012) [19]. All-cause mortality was 43% less in the treatment group (HR: 0.57 (95% CI: 0.41-0.80; p = 0.001) [18]. 

Another recent single-center retrospective study of patients with HFpEF admitted between September 2021 and August 2024, with HF and LVEF of ≥50%, looked at the initiation of SGLT2i while inpatient. Despite a small sample size of 52 patients (25 on SGLT2i and 27 without) who were enrolled, there was significantly higher adherence in terms of continued prescriptions for SGLT2i while discharged from the hospital [19]. Despite this, however, the 30-day and 90-day mortality differences were not statistically significant [19]. Additionally, these groups did not differ in HF exacerbation admissions, all-cause readmissions, or cardiovascular mortality. This retrospective study lacks sufficient power to help generalize these findings to the HFpEF population, showing an area of possible benefit of starting SGLT2i while inpatient for patients with HFpEF. Therefore, an RCT would be needed to better evaluate starting these medications while admitted to better infer morbidity and mortality benefits or a lack thereof. 

Efficacy and safety of dapagliflozin in acute heart failure (DICTATE-AHF) was an open-label RCT of approximately 240 patients with inpatient initiation of dapagliflozin 10 mg within 24 hours of acute HF admission. Approximately 39% of the treatment group patients had HFpEF with LVEF of >50% [20]. Unlike the trials above, the primary outcome of this trial was assessing the efficiency of diuresis with intravenous (IV) furosemide given in conjunction with and without dapagliflozin, measured by cumulative weight change [20]. The primary outcome was not met (OR: 0.65; 95% CI: 0.41-1.02; p = 0.06) [20]. However, dapagliflozin was associated with reduced average doses of furosemide (560 mg with dapagliflozin vs. 800 mg without) (p = 0.006) along with fewer IV Lasix uptitrations (p ≤ 0.05) [20]. The study group with SGLTi showed improved median 24-hour natriuresis (p = 0.03) and urine output (p = 0.005), presumably expediting hospital discharge (Table 2) [20]. Other secondary outcomes measured were not statistically significant from placebo or usual care. These outcomes were as follows: in-hospital worsening HF (p < 0.70), 30-day hospital readmission for acute HF (p < 0.70), change in eGFR (p < 0.70), or HF-related death (p < 0.10) [20]. 

**Table 2:** Overview of trials regarding inpatient initiation of SGLT2i SGLT2i: sodium-glucose cotransporter 2 channel inhibitors; eGFR: estimated glomerular filtration rate; CV: cardiovascular; HF: heart failure; NS: not significant; HR: heart rate; HFpEF: heart failure with preserved ejection fraction; LVEF: left ventricular ejection fraction; T2DM: type 2 diabetes mellitus; KCCQ: Kansas City Cardiomyopathy Questionnaire

Study	Bhatt et al. [15]	Voors et al. [16]	Berg et al. [18]	Lim et al. [19]	Cox et al. [20]
Population & intervention	~1,200 patients with T2DM recently hospitalized with HF; ~21% HFpEF, sotagliflozin or placebo (initiated pre- or within 2 days postdischarge); median follow-up: 9 months	530 patients with acutely decompensated HF; ~22% LVEF >50%; empagliflozin or placebo (initiated within 3 days of stabilization); median follow-up: 90 days	2,401 patients hospitalized for HF; 71.5% LVEF ≤40%; 28.5% LVEF >40%; dapagliflozin or placebo Median follow-up: 2 months	52 patients with HFpEF (LVEF ≥50%) admitted Sep 2021-Aug 2024; 25 on SGLT2i, 27 without; single-center retrospective study	~240 patients admitted for acute HF; ~39% HFpEF (LVEF >50%); dapagliflozin 10 mg or usual care (initiated within 24 hrs of admission); open-label RCT
Primary outcomes	Total cardiovascular deaths and HF hospitalizations significantly reduced with sotagliflozin vs. placebo (HR: 0.67; 95% CI: 0.52-0.85; p < 0.001)	Composite of all-cause death, HF-related events, time to first HF event, and ≥5-point change in KCCQ total symptom score (stratified win ratio 1.36; 95% CI 1.09-1.68; p = 0.0054)	Composite of time to cardiovascular death or worsening HF; no significant difference (HR: 0.86; 95% CI: 0.68-1.08; p = 0.20)	Significantly higher adherence/continued SGLT2i prescriptions postdischarge in the SGLT2i group	Efficiency of diuresis (cumulative weight change with IV furosemide): primary outcome not met (OR: 0.65; 95% CI: 0.41-1.02; p = 0.06)
Secondary outcomes	Total hospitalizations and urgent visits: HR: 0.64 (95% CI: 0.49-0.83); p < 0.001; cardiovascular death: 95% CI: 0.58-1.22; p = 0.36 (NS); KCCQ-12 score at 4 months: 95% CI: 1.3-7.0; p = 0.005; change in eGFR: 95% CI -1.30 to 0.98 (NS)	CV death or HF exacerbation: 12.8% vs. 18.5% (HR: 0.69; 95% CI: 0.45-1.08) (NS) KCCQ at 90 days: adjusted mean difference +4.45 points (95% CI: 0.32-8.59) NT-proBNP at day 30: geometric mean ratio 0.90 (95% CI: 0.82-0.98)	Worsening HF events: 9.4% vs. 10.3% (HR: 0.91; 95% CI: 0.71-1.18) (NS); CV death: 2.5% vs. 3.1% (HR: 0.78, 95% CI: 0.48-1.27) (NS); all-cause death: 3.0% vs. 4.5% (HR: 0.66; 95% CI: 0.43-1.00) (NS)	30-day and 90-day mortality: no significant difference; HF exacerbation admissions: no significant difference; all-cause readmissions: no significant difference; CV mortality: no significant difference	Reduced average furosemide dose (560 mg vs. 800 mg; p = 0.006); fewer IV furosemide up-titrations (p ≤ 0.05); improved 24-hr natriuresis (p = 0.03) and urine output (p = 0.005); in-hospital worsening HF; 30-day readmission, eGFR change, HF-related death: all NS

The evidence supporting the use of SGLT2i in HFpEF has evolved rapidly, anchored by two landmark outpatient trials and supplemented by a growing body of inpatient data. Extrapolating the data from these trials establishes SGLT2i as a cornerstone of HFpEF management and provides evidence favoring their initiation during hospitalization prior to discharge. 

Outpatient evidence: EMPEROR-preserved and DELIVER

EMPEROR-preserved and DELIVER represent the foundational evidence for SGLT2i use in ambulatory HFpEF. Both trials enrolled nearly 6,000 patients each and demonstrated clinically meaningful reductions in the primary composite endpoint of cardiovascular death or HF hospitalization/worsening. Notably, neither trial demonstrated a statistically significant reduction in cardiovascular mortality as an isolated endpoint. It is unclear why this pattern exists, but the data suggest there are competing causes of death in this high-comorbidity-burdened population. These trials did, however, show a robust improvement in KCCQ score, with patients having significantly less symptom frequency, severity, and better quality of life. While the trials showed a statistically significant difference, they fall below the commonly cited five-point minimal clinically important difference for the KCCQ, warranting cautious interpretation of their symptomatic impact at the individual patient level [13]. This is supported by a study by Butler et al. with a KCCQ score improvement of ≥7 points in HFpEF is clinically significant [21]. However, there are studies that contrast this standard, showing clinically meaningful benefits can be achieved with a KCCQ score improvement of 2.5 points corresponding to a number needed to treat (NNT of ~17) [22-23]. 

Inpatient initiation: SOLOIST-WHF, EMPULSE, and DAPA ACT HF-TIMI 68

A critical clinical question beyond the efficacy of SGLT2i in HFpEF is the optimal timing of initiation, specifically, whether in-hospital initiation during an acute decompensation confers additional benefit. A review of four trials discussed earlier alludes to the overall benefit of inpatient initiation vs. outpatient. 

The SOLOIST-WHF trial further supports the beneficial effects of SGLT2i in HFpEF. Although the trial primarily focused on outcomes in patients with more severe HF, its findings underscore the importance of early initiation of SGLT2i in preventing recurrent hospitalizations and urgent HF visits. The lack of significant impact on mortality in this trial aligns with findings from other studies, suggesting that while SGLT2i can mitigate worsening HF and improve symptomatic outcomes, the effect on mortality may require longer follow-up or be more pronounced in specific patient subsets.

The EMPULSE trial included a notable subset with HFpEF and showed improvement in clinical outcomes such as the time to first HF event and the change in KCCQ scores. This trial adds to the growing evidence that early initiation of SGLT2i in HFpEF patients while hospitalized may help prevent further deterioration and improve functional status. Moreover, the stratified analysis within EMPULSE found no significant modification of the primary endpoint by EF, suggesting that the benefits of SGLT2i in HFpEF may be independent of LVEF, further supporting their broad applicability in HF management. 

The DAPA ACT HF-TIMI 68 trial, though primarily focused on patients with HFrEF, included a notable subgroup with HFpEF. Although the trial did not find statistically significant differences in cardiovascular death or worsening HF for this subgroup, the broader evidence from other trials suggests that the effects of dapagliflozin in HFpEF may still offer clinical benefit. The authors of the study believed this finding was likely due to the study being underpowered, a short two-month follow-up period, and lower-than-expected event rates. Overall, this meta-analysis demonstrated a trend toward improved outcomes in patients receiving SGLT2i during hospitalization. 

Overall, the consistent findings across these trials suggest that SGLT2i are not only effective in managing the symptoms and clinical course of HFpEF, but they also improve the overall management of patients with HF by reducing hospitalizations and enhancing quality of life. Although mortality benefits may be less pronounced in HFpEF compared to HFrEF, the significant reduction in hospitalizations and improvement in symptom scores highlight the therapeutic potential of SGLT2i in this challenging population. Future studies, particularly long-term outcomes trials, are needed to further elucidate the impact of SGLT2i on all-cause mortality and to refine treatment strategies for patients with HFpEF. 

## Conclusions

Several limitations merit consideration when interpreting this body of evidence. First, the inpatient trials enrolled mixed HF populations, with HFpEF patients representing a minority (21-28.5%), limiting the ability to draw HFpEF-specific conclusions from these studies. Second, the KCCQ improvements observed across trials, while statistically significant, were generally modest and may not uniformly translate to clinically perceptible symptomatic benefit depending on the literature reviewed. Third, the safety signals observed in DAPA ACT HF-TIMI 68, particularly symptomatic hypotension and worsening renal function, highlight the importance of individualized risk-benefit assessment when initiating SGLT2i during acute decompensation. Finally, the relatively short follow-up periods in the inpatient trials leave unanswered questions regarding the durability of benefit and long-term safety. One other unique limitation is in SOLOIST-WF; the trial ended early because of loss of funding from the sponsor, which limits the conclusion to be drawn because of its abrupt cessation. Future research should aim to clarify the optimal timing of SGLT2i initiation in hospitalized HF patients, identify subpopulations most likely to benefit from early initiation, such as HFpEF or HFrEF, and determine whether the mortality signal observed in the pooled inpatient analysis is reproducible in dedicated, adequately powered trials.

## References

[1] McKee PA, Castelli WP, McNamara PM, Kannel WB (1971). The natural history of congestive heart failure: the Framingham study. N Engl J Med.

[2] Redfield MM, Borlaug BA (2023). Heart failure with preserved ejection fraction: a review. JAMA.

[3] Cannata A, McDonagh TA (2025). Heart failure with preserved ejection fraction. N Engl J Med.

[4] Borlaug BA, Sharma K, Shah SJ, Ho JE (2023). Heart failure with preserved ejection fraction: JACC scientific statement. J Am Coll Cardiol.

[5] Kittleson MM, Panjrath GS, Amancherla K (2023). 2023 ACC expert consensus decision pathway on management of heart failure with preserved ejection fraction: a report of the American College of Cardiology Solution Set Oversight Committee. J Am Coll Cardiol.

[6] Campbell P, Rutten FH, Lee MM, Hawkins NM, Petrie MC (2024). Heart failure with preserved ejection fraction: everything the clinician needs to know. Lancet.

[7] Vahid SS, Jahromi MS, Moukarbel GV, Hill JW (2026). Mechanistic insights and clinical horizons of SGLT2 inhibitors in heart failure management. Trends Cardiovasc Med.

[8] Lytvyn Y, Bjornstad P, Udell JA, Lovshin JA, Cherney DZ (2017). Sodium glucose cotransporter-2 inhibition in heart failure: potential mechanisms, clinical applications, and summary of clinical trials. Circulation.

[9] Zelniker TA, Braunwald E (2020). Mechanisms of cardiorenal effects of sodium-glucose cotransporter 2 inhibitors: JACC state-of-the-art review. J Am Coll Cardiol.

[10] Masuda T, Kariro K, Morishita Y (2026). Why are SGLT2 inhibitors effective in HFpEF? Implications for interstitial fluid retention and cardio-renal interaction. Hypertens Res.

[11] Li X, Preckel B, Hermanides J, Hollmann MW, Zuurbier CJ, Weber NC (2022). Amelioration of endothelial dysfunction by sodium glucose co-transporter 2 inhibitors: pieces of the puzzle explaining their cardiovascular protection. Br J Pharmacol.

[12] Polack FP, Thomas SJ, Kitchin N (2021). Safety and efficacy of the BNT162b2 mRNA Covid-19 vaccine. N Engl J Med.

[13] Spertus JA, Jones PG, Sandhu AT, Arnold SV (2020). Interpreting the Kansas City Cardiomyopathy Questionnaire in clinical trials and clinical care: JACC state-of-the-art review. J Am Coll Cardiol.

[14] Solomon SD, McMurray JJ, Claggett B (2022). Dapagliflozin in heart failure with mildly reduced or preserved ejection fraction. N Engl J Med.

[15] Bhatt DL, Szarek M, Steg PG (2021). Sotagliflozin in patients with diabetes and recent worsening heart failure. N Engl J Med.

[16] Voors AA, Angermann CE, Teerlink JR (2022). The SGLT2 inhibitor empagliflozin in patients hospitalized for acute heart failure: a multinational randomized trial. Nat Med.

[17] Tromp J, Kosiborod MN, Angermann CE (2024). Treatment effects of empagliflozin in hospitalized heart failure patients across the range of left ventricular ejection fraction-results from the EMPULSE trial. Eur J Heart Fail.

[18] Berg DD, Patel SM, Haller PM (2025). Dapagliflozin in patients hospitalized for heart failure: primary results of the DAPA ACT HF-TIMI 68 randomized clinical trial and meta-analysis of sodium-glucose cotransporter-2 inhibitors in patients hospitalized for heart failure. Circulation.

[19] Lim L, Fattal J, Szwak JA, Brown D, Hiruy A (2026). Inpatient SGLT2I initiation and its impact on HFpEF prescription rates and clinical outcomes. Crit Care Med.

[20] Cox ZL, Collins SP, Hernandez GA (2024). Efficacy and safety of dapagliflozin in patients with acute heart failure. J Am Coll Cardiol.

[21] Butler J, Shahzeb Khan M, Lindenfeld J (2022). Minimally clinically important difference in health status scores in patients with HFrEF vs HFpEF. JACC Heart Fail.

[22] Abdel Jawad M, Jones PG, Arnold SV (2025). Interpreting population mean treatment effects in the Kansas City Cardiomyopathy Questionnaire: a patient-level meta-analysis. JAMA Cardiol.

[23] Butler J, Khan MS, Mori C (2020). Minimal clinically important difference in quality of life scores for patients with heart failure and reduced ejection fraction. Eur J Heart Fail.

